# Mortality Risk Prediction by an Insurance Company and Long-Term Follow-Up of 62,000 Men

**DOI:** 10.1371/journal.pone.0005457

**Published:** 2009-05-06

**Authors:** Eric J. G. Sijbrands, Erik Tornij, Sietske J. Homsma

**Affiliations:** 1 Division of Pharmacology, Vascular and Metabolic Diseases, Department of Internal Medicine, Erasmus Medical Center, Rotterdam, The Netherlands; 2 Actuarial Department, Nationale-Nederlanden – ING Group, Rotterdam, The Netherlands; 3 Medical Department, Nationale-Nederlanden – ING Group, Rotterdam, The Netherlands; University of South Florida, United States of America

## Abstract

**Background:**

Insurance companies use medical information to classify the mortality risk of applicants. Adding genetic tests to this assessment is currently being debated. This debate would be more meaningful, if results of present-day risk prediction were known. Therefore, we compared the predicted with the observed mortality of men who applied for life insurance, and determined the prognostic value of the risk assessment.

**Methods:**

Long-term follow-up was available for 62,334 male applicants whose mortality risk was predicted with medical evaluation and they were assigned to five groups with increasing risk from 1 to 5. We calculated all cause standardized mortality ratios relative to the Dutch population and compared groups with Cox's regression. We compared the discriminative ability of risk assessments as indicated by a concordance index (c).

**Results:**

In 844,815 person years we observed 3,433 deaths. The standardized mortality relative to the Dutch male population was 0.76 (95 percent confidence interval, 0.73 to 0.78). The standardized mortality ratios ranged from 0.54 in risk group 1 to 2.37 in group 5. A large number of risk factors and diseases were significantly associated with increased mortality. The algorithm of prediction was significantly, but only slightly better than summation of the number of disorders and risk factors (c-index, 0.64 versus 0.60, P<0.001).

**Conclusions:**

Men applying for insurance clearly had better survival relative to the general population. Readily available medical evaluation enabled accurate prediction of the mortality risk of large groups, but the deceased men could not have been identified with the applied prediction method.

## Introduction

Insurance companies classify applicants according to their mortality risk. The applicant's risk is estimated from the history of disease and the presence of risk factors. Mortality tables of a reference population and algorithms for assessment of risk are based on medical literature (population-based studies, publications of the natural history of diseases, and intervention trials) and public databases [1], [2]. However, it is likely that policy-holders are healthier than patients in medical studies, and in general insurance contracts last much longer than follow-up in medical studies. Moreover, it is impossible to anticipate effects of novel medical interventions during the lifetime of the contract. Assessment of multiple (and competing) risk factors may improve the estimation of the individual risk, and proposals to include genetic information are currently being debated [2]–[6]. Molecular diagnostics may enhance the individual risk assessment and may lead to personalized medicine in the case of treatable disorders. Therefore, it may be expected that genetic testing in the future will improve the accuracy of the underwriting. But major concerns exists that genetic testing leads to discrimination and, therefore, governments have restricted the use of genetic information by insurers [7].

A fundamental principle of life insurance is to guarantee compensation for loss of income or liability in respect to an event that cannot be predicted with certainty. The risk of an individual is allocated to large numbers of people. Improving the discriminative ability of predicting mortality risk may, from this point of view, be undesirable. Whether present-day underwriting has any prognostic value is unknown and this complicates the discussion about improving the risk assessment. For example, the discussion about adding (genetic) tests related to common diseases may prove to be a purely theoretical exercise in case applicants have low absolute mortality risks. It is unlikely that a better prediction method (higher likelihood) will have any relevance in populations with very low risk. Even within families, the mortality from untreated monogenic disorders shows large variation, which reduces the value of molecular diagnostics for the underwriting process [8]. Probably, the burden of a few very rare Mendelian disorders can be well predicted by molecular means, but family history offers the insurer an opportunity to identify health risks and to set, if necessary, specific premiums without extra costs of tests.

We hypothesized that the present-day underwriting has no relevant discriminative ability and improving this risk assessment with additional tests will be difficult, because the majority of applicants is characterized by low risk. Therefore, we compared a simple risk assessment method in male applicants for life insurance related to mortgage, widows' pensions, or income with the observed mortality during long-term follow-up. In addition, we assessed the prognostic value of the risk prediction.

## Methods

### Ethics statement

The study was approved by the Medical Ethics Committee of the Erasmus MC and all applicants gave written informed consent for risk analyses and actuarial studies.

### Subjects

Medical records were available of 62,378 applicants whose mortality risk was estimated because they applied for life insurance (mortgage insurance, widows' pensions, and income protection). The intake of the applicants was performed between June 1, 1962 and June 1, 1990. In 2002, we ended the follow-up. We excluded 44 men because of incompleteness of information resulting in a study population of 62,334 men. The data were initially stored on punch cards, and a number of electronic databases were used thereafter. The databases were merged and anonymized for the present study.

### Risk assessment

The medical officers performed risk classifications by calculating the mortality ratio relative to reference populations using age, duration of the contract, history of common diseases, and the presence of risk factors [1], [2]. All applicants completed a health questionnaire. Weight and length were measured and a body mass index was calculated. Unfortunately, only obesity was documented in the database: obesity was tagged when the body mass index was above 30 kg/m^2^. Medical examination was performed when the medical officer required additional medical information on indication and when the average insured amount was equal to or above $45,000, which gradually increased during 28 calendar years up to $65,000. Serum glucose and urinary tests for glucose and albumin were included; HIV-testing was added in 1987. Screening of serum total and high-density lipoprotein cholesterol levels was added in 1990. In Table 1, only the most frequent disorders (above 0.5 percent) are shown, though the full medical history of each applicant was obtained.

**Table 1:** The most frequent findings in a Dutch male cohort and the association with mortality within this cohort.

Risk Factor	n	Percentage (95% CI)	Relative risk (95% CI)	P-value
Cardiovascular risk factors				
Angina pectoris, myocardial infarction, CABG	1034	1.7 (1.6–1.8)	2.23 (1.90–2.62)	<0.001
Other cardiovascular disorders	2927	4.7 (4.5–4.9)	1.20 (1.04–1.38)	0.01
Family history of cardiovascular disease	3116	5.0 (4.8–5.2)	1.20 (1.01–1.42)	0.04
Blood pressure above 145/90 mmHg*	8414	13.5 (13.2–13.8)	1.55 (1.43–1.69)	<0.001
Hypertension†	1691	2.7 (2.6–2.8)	1.66 (1.36–2.04)	<0.001
History of cholesterol above 5.5 mmol/L	1246	2.0 (1.9–2.1)	1.14 (0.87–1.49)	0.4
Diabetes mellitus (onset before age 30)	429	0.7 (0.6–0.8)	3.27 (2.32–4.62)	<0.001
Diabetes mellitus (onset on or after age 30)	647	1.0 (0.96–1.1)	2.08 (1.61–2.53)	<0.001
Body mass index above 30 kg/m^2^	1471	2.4 (2.2–2.5)	1.04 (0.73–1.49)	0.8
Smoking of more than 20 cigarettes daily	5081	8.2 (7.9–8.4)	1.54 (1.39–1.72)	<0.001
Other risk factors				
Alcohol consumption above 60 g daily	2019	3.2 (3.1–3.4)	1.38 (1.17–1.61)	<0.001
Chronic obstructive pulmonary disease	2378	3.8 (3.7–4.0)	1.55 (1.29–1.85)	<0.001
Psychiatric disorder	6548	10.5 (10.3–10.7)	1.13 (1.01–1.27)	0.04
Epilepsy	816	1.3 (1.2–1.4)	2.14 (1.62–2.84)	<0.001
Malignancies	798	1.3 (1.2–1.4)	1.78 (1.39–2.29)	<0.001
Low back pain	2201	3.5 (3.4–3.7)	0.54 (0.35–0.83)	0.005
Osteoarthritis, rheumatic disorders	490	0.8 (0.7–0.9)	1.72 (1.27–2.33)	<0.001
Herniated vertebral disc	1325	2.1 (2.0–2.2)	0.95 (0.75–1.21)	0.7

Cox's regression analysis was performed with adjustment for year of birth and age at medical evaluation.

CABG denotes coronary artery by-pass grafting; CI denotes confidence interval; other cardiovascular disorders were atrial fibrillation, heart failure, peripheral vascular disease, cardiomyopathy, subarachnoid hemorraghe, cerebrovascular accident, valve disease, and congenital disorder.

* measurement of blood pressure performed by the general practitioner or the medical officer during a single office visit.

† hypertension was diagnosed by general practitioner or specialist and treatment with antihypertensive medication was started.

Individual mortality risk was predicted by summation of risk factor specific percentages according to guidelines that were made by the actuarial and medical departments of Nationale-Nederlanden. The risk factor specific percentages in these guidelines were based on life expectancies in long term clinical studies and life tables of the general population [1], [2], [8]–[10]. During the years, the risk factor specific percentages were adjusted according to changes in mortality of the reference population and/or changes in life expectancy resulting from new medical treatments. The most important changes were based on adjustments for diabetes mellitus, hypertension, and survival after coronary artery bypass grafting [11]–[18]. The risk of each client was estimated and expressed as percentage. After summation of the excess mortalities related to risk factors and diseases the medical officer assigned the client to one of five risk groups: applicants without overt risk factors were assigned to group 1 (100 percent); applicants with an estimated risk 101–130 percent to group 2; 131–200 percent to group 3; 201–400 percent to group 4; and above 400 percent to group 5. Tables of risk factor and disease specific death rates were provided in guidelines made by the actuarial and medical departments of Nationale-Nederlanden. The expected deaths were estimated by the actuary with age and male specific annual death rates of the Dutch Central Bureau of Statistics (CBS). The total expected deaths of persons with age x(0) obtaining insurance at t = 0 for the duration of n years is estimated with
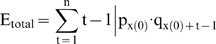
in which p_x(0)_ is the probability that (x) will live one year and q_x(0)_ is the probability that (x) will die within n year(s). The death rates observed by CBS were used as q_x(0)_. The year starts at t−1 and ends at t. We need to consider the survival probabilities (p_x(0)_), because during long follow-up and/or in case of disorders with large excess mortality the p_x(0)_ becomes substantially smaller than 1. For example a male client of 58 years with epilepsy, who applied for life insurance in 1965, had an annual death rate of 10% in the CBS statistics. The client had a 49% death rate during 7 years (and not 7.10 = 70%). Relative to group 1, a mortality risk of 232% was estimated assigning the client to group 4.

### Statistical analyses

All cause mortality was compared with the mortality in the male Dutch population standardized for age and calendar period as described previously [8], [19]. Briefly, the standardized mortality ratio is the ratio of observed to expected number of deaths. We calculated the expected mortality by multiplying the total number of years lived by the men in each calendar period for each age category by the age and male specific mortality rates of the Dutch population for each calendar period. We compared mortality between subgroups with Cox's regression (relative risk). Cumulative survival was analyzed with the Kaplan-Meier method. We censored participants when they died or reached the end of the insurance contract (on December 31, 2002 the last contracts ended). We calculated the 95 percent confidence interval of the standardized mortality ratio assuming a Poisson distribution of the observed number of deaths and using exact limits. We calculated the 95 percent confidence interval of the relative risk with Cox's regression as the exponent of the regression coefficient and its standard error. Significance was assessed at the 5 percent level of probability.

The discriminative ability of predictive models was evaluated by comparing the concordance indices (c) using S-plus for Windows 2000 [20]. The concordance statistic for survival data indicates the probability that for a randomly chosen pair of persons, the one having the higher predicted survival is the one who survives longer: 0.5 indicates no discriminative ability and 1.0 indicates no false classifications. An internal validation was performed by taking 100 random bootstrap samples [21].

## Results

We have analyzed data on 62,334 males with a mean follow-up of 13 (SD±9) years (median 12; range 4 months to 41 years). The medical evaluation was performed at a mean age of 37 (SD±10; median 36) years ranging from 18 to 77. Table 1 shows the most frequent baseline medical findings.

During follow-up, a total of 3,433 deaths were observed in 844,815 person years. The all cause mortality relative to the Dutch male population standardized for age and calendar period was 0.76 (95 percent confidence interval, 0.73 to 0.78, P<0.001).

**Figure 1:**
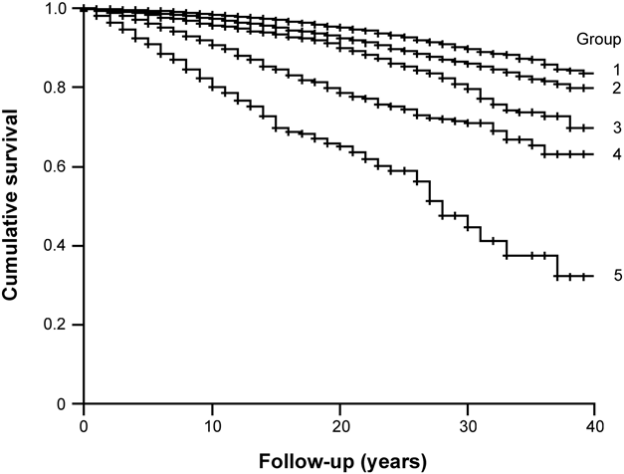
Survival during follow-up in a large male cohort according to *a priori* risk prediction. The Kaplan-Meier curves show a significant association between predicted risk and observed life expectancy.

The standardized mortality ratio according to predicted risk is shown in Table 2. Risk groups 1 and 2 had a better life expectancy than the Dutch population. The mortality risk of group 3 was similar to that of the Dutch population. Significant excess mortality relative to the Dutch population was observed in risk groups 4 and 5. The standardized mortality ratio of group 5 was 2.37 (95 percent confidence interval, 2.04 to 2.75, P<0.001). We also performed direct comparisons using risk group 1 as a reference group in a Cox's model with adjustment for year of birth and age at medical evaluation. Group 2 had 1.34 (95 percent confidence interval, 1.23 to 1.46), group 3 had 1.87 (95 percent confidence interval, 1.69 to 2.07), group 4 had 3.03 (95 percent confidence interval, 2.68 to 3.43), and group 5 had 4.64 (95 percent confidence, 3.90 to 5.53) times increased mortality risk relative to group 1 (all P<0.001). Figure 1 shows the difference in cumulative survival between the risk groups (Log-rank test P<0.001).

**Table 2:** Mortality in a large male cohort compared to the Dutch population according to *a priori* risk classification.

Risk group	No of men	Person years	No of deaths	Standardized mortality ratio (95% confidence interval)
1	24,666	358,296	942	0.54 (0.50 to 0.57)
2	22,137	302,857	1,185	0.70 (0.66 to 0.74)
3	11,164	138,841	739	0.96 (0.89 to 1.03)
4	3,469	36,728	389	1.57 (1.41 to 1.73)
5	898	8,092	178	2.37 (2.04 to 2.75)
Total group	62,334	844,815	3,433	0.76 (0,73 to 0.78)

The associations between specific baseline findings and mortality during follow-up were determined with Cox's regression adjusted for the year of birth and age at medical evaluation (Table 1). Coronary artery disease (angina pectoris, myocardial infarction, and coronary by-pass grafting), other cardiovascular disorders (atrial fibrillation, heart failure, peripheral vascular disease, cardiomyopathy, subarachnoid hemorrhage, cerebrovascular accident, valve disease, and congenital disorders), a positive family history of cardiovascular disease, elevated blood pressure during a single office visit, hypertension, diabetes mellitus, smoking more than 20 cigarettes daily, consuming more than 60 g alcohol daily, chronic obstructive pulmonary disease, psychiatric disorders, epilepsy, malignancies, osteoarthritis and rheumatic disorders were all significantly associated with an increased mortality risk. Obesity was found in 2.4 percent (95 percent confidence interval, 2.2 to 2.4 percent) of the applicants and was not associated with excess mortality. Those with obesity had significantly more often hypertension (4.9 versus 2.7 percent, p<0.001) and tended to have more often type 2 diabetes (2.0% versus 1.0%, p = 0.09) compared to non-obese clients. The serum total cholesterol concentration was not routinely measured during the medical evaluation and the information about hypercholesterolemia was only available in a minority of the client's histories. In this subgroup, hypercholesterolemia was not associated with an increased mortality risk. Low back pain and herniated vertebral disc did not contribute to mortality. In fact, low back pain was associated with a low relative mortality risk (0.54, 95 percent confidence interval, 0.35–0.83). Obese persons had more often low back pain than non-obese (5.3 versus 3.5 percent, p<0.001). In 886 person years, we did not observe deaths among 78 obese persons with low back pain while the expected standardized rate was 3 (expected number of deaths).

The risk of one up to the combination of three disorders relative to the group without baseline disorders increased in a dose dependent manner: 1.45 up to 2.39 (see Table 3).

**Table 3:** The observed mortality relative to combinations of disorders and risk factors that were documented at baseline.

Number of Disorders and/or Risk Factors	n	Percentage (95% CI*)	Relative risk (95% CI*)	P-value
subjects without finding	9,594	15.4 (15.1–15.7)	1.00	
subjects with one finding	35,150	56.4 (56.0–56.8)	1.45 (1.29–1.62)	<0.001
subjects with two findings	13,594	21.8 (21.5–22.1)	1.76 (1.56–1.98)	<0.001
subjects with three findings	3,996	6.4 (6.2–6.6)	2.39 (2.08–2.75)	<0.001

Cox's regression analysis was performed with adjustment for year of birth and age at medical evaluation.

* CI denotes confidence interval.

In all Cox's regression analyses, the year of birth had a significant inverse relation with mortality (relative risk 0.97, 95 percent confidence interval, 0.96 to 0.98). Age was positively associated with mortality (relative risk 1.06, 95 percent confidence interval, 1.05 to 1.07).

We have estimated c-indices to evaluate the accuracy of the following risk prediction methods: the individual risk predictions and the classification in 5 risk groups had virtually identical c-indices (0.64, 95 percent confidence interval, 0.63 to 0.65). The actuarial risk predictions were significantly more accurate than simple counting of disorders and risk factors (P<0.001). The c-index of just counting the number of disorders was 0.60 (95 percent confidence interval, 0.59 to 0.61).

## Discussion

We have analyzed a large male cohort with long-term follow-up and we have found that the classification of the men according to mortality risk correlated with the observed mortality. Insurance companies often request medical information and our results suggest that relatively simple information can be used to make accurate risk assessments of groups. The risk predictions are needed to substantiate risk specific premiums and to avoid acceptance at standard risk of persons with very short life expectancy. Nonetheless, the deceased men in our cohort could not have been identified individually by the limited medical evaluation.

In general, severely ill and disabled persons do not apply for insurance. Hence, as expected, we observed selection of healthy individuals. Although the cohort had a better life expectancy compared to the general Dutch population, we were able to observe a large variation of mortality and clear excess mortality in risk groups 4 and 5. Cardiovascular diseases and cardiovascular risk factors, especially diabetes mellitus, strongly contributed to variation of mortality risk. Neurologic disorders, malignancies, disorders with arthritis, and chronic obstructive pulmonary disease were also associated with an increased risk of death. Although the present population had a better overall life expectancy, the mortality risk estimates of the disorders and risk factors were in accordance with those in population-based studies [8]–[10]. As expected, low back pain did not contribute to mortality and was even associated with improved prognosis! To our knowledge, this is the first report of an inverse association between low back pain and mortality. It is tempting to hypothesize that low back pain leads to risk avoidance [22].

Our results support that simple risk assessment is accurate to set the price of the premium for groups of applicants and suggests that additional tests result in extra costs without clear benefits. The low absolute risk of the majority of applicants suggests that additional (for instance, genetic) tests would need to provide a level of prediction higher than currently achievable to improve the individual risk prediction.

Risk prediction is necessary to estimate the costs of insurances and to calculate optimal premiums. The final premium will be influenced by market demands and competition among insurance companies as well as medical and ethical issues determining the choice between risk specific premiums and generalizable risk assignment. Although an insurance company may not be interested in (investing in) medical information from applicants, it may still need to consider other factors as a result of clients' demands. Non-smokers may refuse to pay for smokers' risk. Thin clients may not want to pay for the risk of obesity. Clients may ask for reduced premiums as a reward for a healthy lifestyle and a favourable genotype. Such public demands for predictive power of medical evaluations may result in highly risk-dependent insurance premiums. Prediction of individual risk may be improved by adding multiple risk factors such as levels of apolipoproteins A1 and B, lipoprotein(a), high-density lipoprotein cholesterol, homocysteine, tumour markers, and genetic information [2], [23], [24]. However, in a low risk population, the extra costs of these tests are solely intended to avoid selection of adverse risk.

Obviously the strength of the present study lies in the long-term prospective follow-up of a relatively healthy population and the availability of excellent general population statistics enabling reliable analyses of all cause mortality: prospective follow-up of long lasting insurance contracts resulted in a unique historical cohort and enabled assessment of the absolute risk. Our study offers a basis for discussions about improving risk prediction by insurers and whether or not additional tests are useful [25]. A weakness is the lack of complete information on a number of risk factors because their importance was not yet apparent in 1962. For example, we had blood pressure measurements from only a single office visit, whereas the seventh Joint National Committee (JNC 7) proposed a definition of hypertension based on the average of two or more visits [26]. Nevertheless, we found that increased blood pressure during a single office visit was clearly associated with excess mortality, confirming findings of others [27]. Remarkably, treated hypertension was still associated with excess mortality. This may be explained by a late diagnosis, lack of compliance, or insufficient treatment. We found severe excess mortality among clients with type 1 and insulin dependent type 2 diabetes. During more recent years, the prognosis of patients with type 1 diabetes may have improved considerably [28]. Moreover, Japanese patients had greater excess mortality than Finnish patients with type 1 diabetes [29]. Hence, our estimates may not apply to the present-day male patient with diabetes mellitus and may not be generalized to populations in other countries. Obesity was not associated with excess mortality, but we lack insight in the exact contrast between the obese and non-obese group. Body mass indexes were not available in the database, because only obesity (body mass index equal to or above 30 kg/m^2^) was tagged in the database. Although obesity was rare and only available as dichotomous variable in our historical cohort, obese persons had more often hypertension and tended to a higher frequency of type 2 diabetes. A limited number of applicants had information about previous cholesterol measurements and, in an even smaller group, total cholesterol was measured during the medical evaluation. Hypercholesterolemia was not associated with increased mortality reflecting the lack of sufficient information.

We conclude that medical evaluation by an insurance company enables accurate prediction of the mortality risk of large groups of men. Cardiovascular and neurologic diseases, malignancies, arthritis, and chronic obstructive pulmonary disease were associated with an increased mortality risk. However, decedents could not have been identified individually by the medical evaluation employed. The low mortality risk of our population suggests that individual risk prediction is unlikely to achieve relevant post-test probabilities, which is in support of equal opportunities for those who are seeking life insurance.
